# Cardiac β‐adrenergic receptor activation mediates distinct and cell type‐dependent changes in the expression and distribution of connexin 43

**DOI:** 10.1111/jcmm.15469

**Published:** 2020-06-24

**Authors:** Yi Zhang, Meng‐Chen Hou, Jing‐Jing Li, Ying Qi, Yu Zhang, Gang She, Yu‐Jie Ren, Wei Wu, Zheng‐Da Pang, Wenjun Xie, Xiu‐Ling Deng, Xiao‐Jun Du

**Affiliations:** ^1^ Department of Physiology and Pathophysiology School of Basic Medical Sciences, Xi’an Jiaotong University Health Science Center Xi’an China; ^2^ Department of Pathology Xi’an Guangren Hospital Xi’an Jiaotong University Health Science Center Xi’an China; ^3^ The Key Laboratory of Biomedical Information Engineering of Ministry of Education School of Life Sciences and Technology Xi’an Jiaotong University Xi'an China; ^4^ Key Laboratory of Environment and Genes Related to Diseases Ministry of Education Xi’an Jiaotong University Health Science Center Shaanxi China; ^5^ Experimental Cardiology Laboratory Baker Heart and Diabetes Institute Melbourne Australia

**Keywords:** cardiac fibrosis, connexin 43, gap junction remodelling, IL‐18, β‐AR activation

## Abstract

Activation of the sympatho‐β‐adrenergic receptors (β‐ARs) system is a hallmark of heart failure, leading to fibrosis and arrhythmias. Connexin 43 (Cx43) is the most abundant gap junctional protein in the myocardium. Current knowledge is limited regarding Cx43 remodelling in diverse cell types in the diseased myocardium and the underlying mechanism. We studied cell type‐dependent changes in Cx43 remodelling due to β‐AR overactivation and molecular mechanisms involved. Mouse models of isoproterenol stimulation or transgenic cardiomyocyte overexpression of β_2_‐AR were used, which exhibited cardiac fibrosis and up‐regulated total Cx43 abundance. In both models, whereas Cx43 expression in cardiomyocytes was reduced and more laterally distributed, fibroblasts exhibited elevated Cx43 expression and enhanced gap junction communication. Mechanistically, activation of β_2_‐AR in fibroblasts in vitro elevated Cx43 expression, which was abolished by the β_2_‐antagonist ICI‐118551 or protein kinase A inhibitor H‐89, but simulated by the adenylyl cyclase activator forskolin. Our in vitro and in vivo data showed that β‐AR activation‐induced production of IL‐18 sequentially stimulated Cx43 expression in fibroblasts in a paracrine fashion. In summary, our findings demonstrate a pivotal role of β‐AR in mediating distinct and cell type‐dependent changes in the expression and distribution of Cx43, leading to pathological gap junction remodelling in the myocardium.

## 1 INTRODUCTION

Activation of β‐adrenergic receptors (β‐ARs) by catecholamines released from sympathetic nerves maintains cardiac output to meet the increased metabolic need.^1^ However, sustained stimulation of β‐ARs is usually associated with notable cardiac pathology.^2^, ^3^ Indeed, rodents subjected to β‐adrenergic receptors overstimulation by pharmacological (eg isoproterenol and ISO) or genetic means (ie mice with transgenic overexpression of β_1_‐ or β_2_‐AR) develop myocardial dysfunction, remodelling, cardiomyocyte loss or hypertrophy, fibrosis and ventricular arrhythmias.^4^, ^5^, ^6^ In the diseased myocardium, β_1_‐ARs undergo down‐regulation and desensitization whilst β_2_‐ARs are redistributed across cardiomyocyte membrane mediating a more widespread signalling.^7^, ^8^, ^9^, ^10^, ^11^ Nearly, all cardiac cell types are equipped with β‐ARs; whereas β_1_‐AR and β_2_‐AR are major subtypes of β‐ARs in cardiomyocyte,^12^, ^13^ only β_2_‐AR presents on fibroblasts or inflammatory cells.^14^, ^15^

Electrical coupling of adjacent cardiomyocytes is critical for coordinated cardiac function. This is achieved through intercellular transportation of ionic currents and small molecules *via* gap junctions that are composed of connexin channels and mainly localized to the intercalated discs (ID) of cardiomyocytes.^16^ Disorganizations of gap junction have been regarded to contribute to cardiac arrhythmias and dysfunction.^17^ In adult mammalian ventricle, connexin 43 (Cx43) is the most abundant isoform of gap junctions. Using myocardial biopsies from patients with hypertrophic, dilated, arrhythmic or ischaemic cardiomyopathies or animal models, numerous studies have shown suppressed Cx43 expression with reduced coupling between cardiomyocytes.^17^ Such change might result in reduced conduction velocity between cardiomyocytes rendering the heart more susceptible to re‐entry.^18^, ^19^ In a mouse model of cardiac‐restricted Cx43 gene deletion, animals exhibited overt slow conduction velocity and arrhythmic death without evident fibrosis.^20^ Although some studies indicated that genetic Cx43 up‐regulation or pharmacological enhancement of intercellular communication via gap junctions is anti‐arrhythmic,^21^, ^22^ there are also studies reporting excessive expression of Cx43 in arrhythmic animal models ^4^, ^23^, ^24^ or in atrial biopsy of patients with atrial fibrillation.^25^, ^26^ In these studies, a robust interstitial fibrosis is a common feature of the myocardium, suggesting that myocardial fibrosis could affect Cx43 expression and localization. In this context, it is essential to illustrate the role of well‐known pro‐fibrotic factors, including catecholamines, in regulating expression and distribution of Cx43.

In the present study, we aimed to examine the changes of Cx43 in cardiomyocytes and fibroblasts induced by β‐AR overactivation, and explore underlying cellular and molecular mechanisms. To achieve this, we employed in vivo mouse models of ISO stimulation or transgenic β_2_‐AR overactivation, and cultured cardiomyocytes or fibroblasts in vitro.

## 2 METHODS

### 2.1 Animals

The β_2_‐AR transgenic (β_2_‐TG) overexpression mouse model was originally developed by Milano *et al* ^27^ Male β_2_‐TG mice and non‐transgenic (NTG) littermates of 3‐ and 5‐months of age were studied. Wild‐type male mice were obtained from Charles River Laboratories. Both strains were on the same C57BL/6 background. Animals were housed in a pathogen‐free environment under a 12/12 hours light‐dark cycle and fed rodent diet ad libitum. All protocols and experimental procedures were approved by the Institutional Animal Care and Use Committee of Xi'an Jiaotong University and conformed to the Guide for the Care and Use of Laboratory Animals published by the National Institutes of Health.

### 2.2 Mouse model of ISO‐induced cardiac fibrosis and drug treatment

Wild‐type male mice (12 week‐old) received ISO (5 mg/kg/day, s.c., #I5627, Sigma‐Aldrich) or saline for 7 days through an osmotic minipump (ALZET model 2001, Cupertino, CA) subcutaneously implanted under isoflurane anaesthesia.

At the end of the 7‐day treatment, the minipump was removed to allow for a period of 12 hours to washout ISO. Then, animals were anaesthetized using isoflurane inhalation for echocardiography using Philip iE33 ultrasound system with a 15‐MHz probe. Short‐axis M‐mode images of the left ventricle were obtained, and colour Doppler‐guided ventricular diastolic filling flows were determined. At the end, animals were killed by anaesthesia overdose and organ weights were measured.

To test the role of β_1_‐ and β_2_‐AR, mice were injected with selective β_1_‐AR antagonists bisoprolol (BIS, 3 mg/kg/day i.p., #B2185, Sigma‐Aldrich) or β_2_‐AR selectively antagonists ICI‐118551 (ICI, 3 mg/kg/day, i.p., #I127, Sigma‐Aldrich) or vehicle (saline) daily for 7 days starting from ISO infusion.

To test the effect of blocking IL‐18 in vivo on myocardium Cx43 expression and redistribution, in mice receiving 7‐day ISO stimulation (5 mg/kg/day), the IL‐18 neutralizing monoclonal antibodies (#BE0237, BioXcell) or control rat IgG (#BE0251, BioXcell) were intraperitoneally injected daily for 7 days starting from ISO infusion.

### 2.3 Sample collection

Heart and blood samples were collected from mice. Euthanasia procedure involves anaesthetization with inhalation of isoflurane (2% in O_2_), blood collection through cardiac puncture followed by cervical dislocation. The heart was quickly dissected for further assays.

### 2.4 Cytokine measurements

Left ventricle (LV) was harvested and snap‐frozen in liquid nitrogen. Tissues were homogenized in phosphate‐buffered saline containing protease inhibitors. IL‐18 levels in LV tissue and plasma were determined by ELISA kit (#ab216165, Abcam). All values were in the linear range of IL‐18 standard curve. Tissue content of IL‐18 was calculated based on protein concentration.

### 2.5 Histology and immunostaining

Histology and immunostaining of heart sections were performed as previously described.^28^ In brief, LV sections were incubated overnight at 4°C with anti‐Cx43 (#3512, Cell Signaling Technology), anti‐α‐SMA (#A5228, Sigma‐Aldrich), anti‐IL‐18 (#ab71495, Abcam), anti‐CD68 (#sc‐20060, Santa Cruz), anti‐α‐actinin (#ab68167, Abcam), anti‐VE‐cadherin (#sc‐9989, Santa Cruz), wheat germ agglutinin (WGA) (#L4895; Sigma‐Aldrich) or Isolectin GS‐IB4 (Alexa Fluor® 488 conjugate, #I21411, Invitrogen) followed by secondary goat anti‐mouse IgGAlexa Fluor® 594 (ab150116, Abcam) and goat anti‐rabbit IgG Alexa Fluor® 555 (ab150078, Abcam). Images were acquired with Leica TCS SP8 STED 3X confocal microscopy. For immunohistochemistry (IHC) staining, light microscopic images were acquired from the myocardium sections using a Zeiss AxioObserver Z1 microscope.

Quantification of Cx43‐positive staining was performed with ImagePro analyser 7.0 software (Media Cybernetics). In blinded fashion, ten randomly selected LV histological areas (20× magnification) were investigated per heart. From each selected field, Cx43‐positive puncta pixel area was obtained and expressed as a ratio of Cx43‐positive area to section area, and the average of ten fields was used as the value per heart. Lateralized Cx43 puncta were chosen when localized on the side of cardiomyocyte membranes or parallel with long axis of cardiomyocyte nucleus, whilst ID Cx43 puncta were on edge of cardiomyocyte or transversely to the long axis of cardiomyocyte nucleus. Summation of the total lateralized pixel was divided by the total transverse pixel and expressed as the ratio of lateralized/ID Cx43.

Quantification of capillary density was performed with ImagePro analyser 7.0 software (Media Cybernetics). Ten randomly myocardial cross‐sectional areas (20× magnification) were investigated per heart. The number of cardiomyocyte and IB4‐labelled microvessels were counted separately and blindly, and the ratio of capillaries to cardiomyocytes was calculated.

### 2.6 Preparation and culture of adult mouse cardiomyocytes

Cardiomyocytes were isolated from adult mouse hearts according to a modified protocol as we previously described.^28^, ^29^ Primary cardiomyocytes in culture were stimulated with ISO (1 μmol/L) for 48 hours. For blockage of β_1_‐ or β_2_‐AR,^30^ bisoprolol (0.1 μmol/L) or ICI‐118551 (0.1 μmol/L) were added to the culture dishes for 30 minutes prior to addition of ISO (1 μmol/L). Proteins were extracted from isolated cardiomyocyte in RIPA buffer containing protease inhibitors and used for Western blotting.

### 2.7 Preparation of adult mouse cardiac fibroblasts

Ventricular fibroblasts were isolated from adult mouse hearts as we previously described.^31^ Fibroblasts were cultured in Dulbecco's modified Eagle's medium (DMEM, 5 mmol/L glucose, Gibco) with addition of 10% foetal bovine serum (FBS, Gibco), penicillin (100 U/mL) and streptomycin (100 μg/mL). Fibroblasts were stimulated with ISO (1 μmol/L), IL‐18 (10 ng/mL, #767004, Biolegend, 48 hours) or Forskolin (100 μmol/L, #F6886, Sigma‐Aldrich) for 24 hours. In some experiments, cells were pre‐treated for 30 minutes with ICI118551 (0.1 μmol/L), H‐89 (0.1 μmol/L, #ab143787, Abcam), respectively, then with ISO treatment for 48 hours.

### 2.8 Dye transfer assay

Lucifer yellow dye transfer assay allows for detection of intercellular dye transportation through gap junction.^32^ Confluent adult mouse fibroblasts were scraped using a syringe needle and then incubated in PBS containing 0.5% lucifer yellow CH, dilithium salt (#861502, Sigma‐Aldrich) for 25 minutes at 37°C. This protocol allows loading of the membrane impermeable dye into damaged cells, followed by intercellular dye transportation *via* gap junctions. Lucifer yellow dye coupled fibroblasts were washed three times with PBS, fixed with 4% paraformaldehyde and photographed using a Leica TCS SP8 STED 3X confocal microscopy. Quantification of positive area was measured with ImagePro analyser 7.0 software (Media Cybernetics).

### 2.9 Western blotting

Western blotting was performed as previously reported.^28^ Polyvinylidene fluoride membranes were probed with primary antibodies against collagen I (#ab34710, Abcam), collagen III (#ab7778, Abcam), Cx43, IL‐18 or GAPDH (ab181602, Abcam), respectively, followed by incubation with the appropriate HRP‐conjugated secondary antibody. All immunoblots were developed using an enhanced chemiluminescence detection system (Bio‐Rad, Hercules, CA). The intensity of bands was quantified using ImageJ software.

### 2.10 Statistics

Data are expressed as mean ± SEM. GraphPad Prism software (version 6.0, GraphPad Software) was used for all statistical analyses. Normality and equal variance were tested followed by statistical analysis using an unpaired two‐tailed Student's* t* test (for two groups) and one‐way ANOVA with Tukey's multiple comparison tests (for groups of three or more). Differences were considered statistically significant at *P* < .05. All tests were 2‐sided.

## 3 RESULTS

### 3.1 β‐AR activation was required for ISO‐induced cardiac remodelling and Cx43 alterations

Echocardiography was performed, and organ weights were measured in mice at the end of a 7‐day period of ISO treatment. In addition to a 10% increase in LV weight indicating hypertrophy, early cardiac decompensation was indicated by moderate degree of LV dilatation and decline in fractional shortening (Table S1). Coronary microvascular dysfunction, indicated by reduced capillary density, is an important feature of hypertrophic cardiomyopathy.^33^ VE‐cadherin immunohistochemical staining of myocardial capillaries revealed no significant alteration in capillary density in LV myocardium from ISO‐treated mice compared to control group (Figure S1A,B).

**Figure 1:**
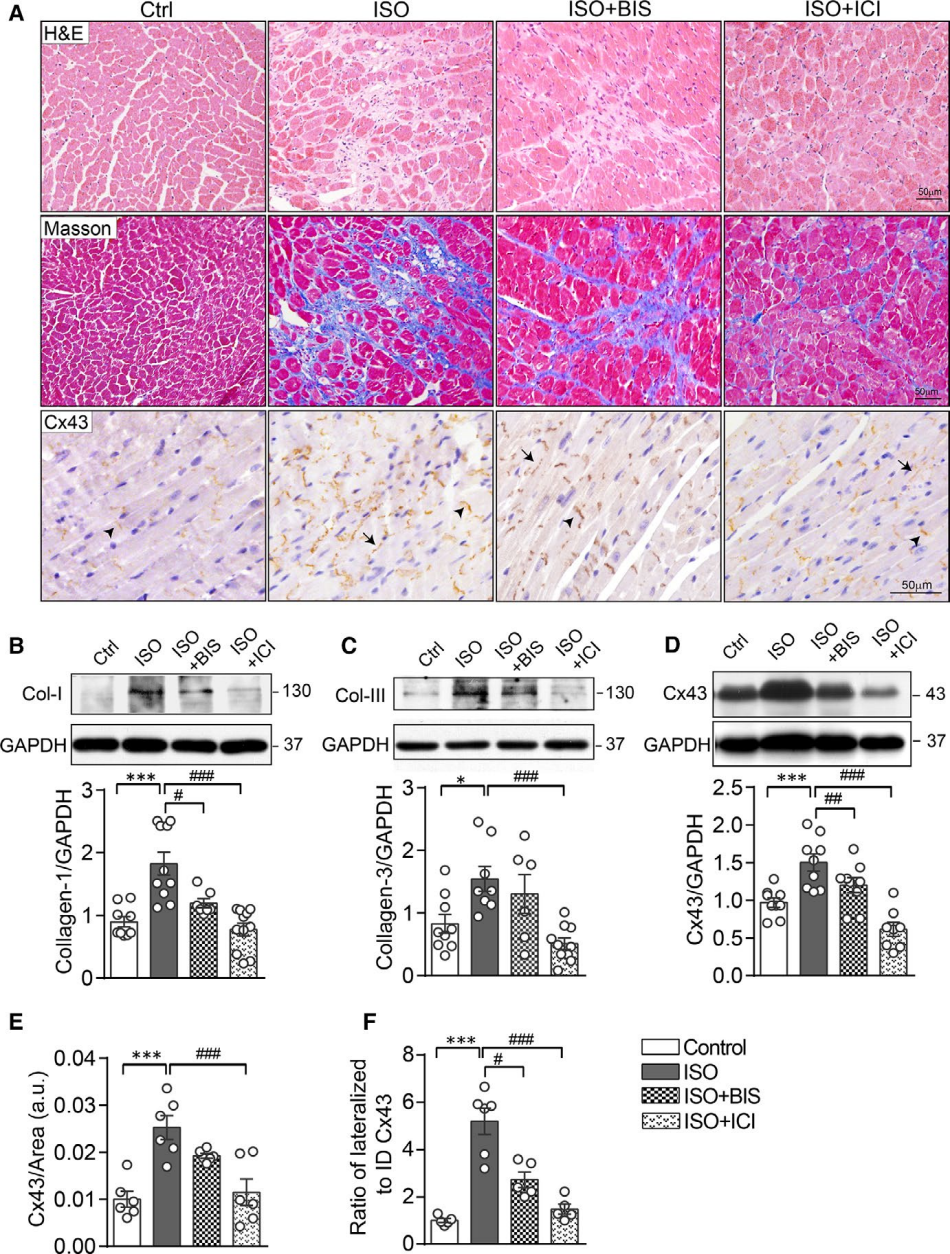
β‐AR activation was required for ISO‐induced cardiac remodelling, Cx43 up‐regulation and redistribution. A, Representative HE staining (upper panels), Masson's trichrome staining (middle panels) and Cx43 immunohistochemistry (IHC) staining (lower panels) of LV myocardium from mice treated with saline (Ctrl), ISO and ISO plus BIS or ICI at day‐7 after treatment. Scale bar: 50 µm. Arrows indicate lateralized Cx43 and arrowheads for ID Cx43 localization. Western blotting images and quantification of band intensity for protein levels of (B) Col‐I, (C) Col‐III and Cx43 (D) in the LV myocardium at day 7 after drug treatment (n = 7‐9 mice/group). Quantificational analysis in IHC staining for (E) Cx43‐positive area and (F) the ratio of lateral to ID localized Cx43 area in LV sections of mice treated with saline, ISO and ISO plus BIS or ICI for 7 d (n = 5‐6 mice/group). Data were expressed as mean ± SEM. *P < .05, **P < .01 and ***P < .001 vs control, ^#^ *P* < .05, ^##^ *P* < .01 and ^###^ *P* < .001 versus ISO. Statistical significance was determined by one‐way ANOVA followed by Tukey's multiple comparisons test

Pathological cardiac remodelling in ISO‐treated mice was evident assessed histologically by HE and Masson's trichrome staining and chemically by expressions of collagen I (Col‐I) and collagen III (Col‐III) (Figure 1A upper and middle panel, B and C). Myocardial fibrosis induced by ISO was reduced by blockade of β_2_‐AR, whilst blockade of β_1_‐AR was less effective (Figure 1A middle panel, B and C).

We examined ISO‐mediated changes in Cx43 expression by Western blotting analysis of LV lysate. Compared with control group, LVs from ISO‐treated mice showed up‐regulated expression level of Cx43 (Figure 1D). Correspondingly, immunohistochemical staining in LV sections showed approximately 2.5‐fold increment in Cx43‐positive area by ISO stimulation (Figure 1A lower panel and E). Cx43 redistribution in cardiomyocytes was also observed throughout the ISO‐treated hearts that was determined by a 5‐fold increase in the ratio of lateral borders vs ID localized Cx43‐positive puncta (black arrow indicated) (Figure 1A lower panel and F). These changes in expression and lateralization of Cx43 in ISO‐treated LV were reversed by administration with ICI‐118551, but bisoprolol was less effective (Figure 1A lower panel and E, F).

### 3.2 β_2_‐AR activation enhanced Cx43 expression and intercellular coupling in fibroblasts

Immunofluorescence staining of Cx43 with cell type‐specific labels displayed that in LV myocardium from ISO‐treated mice, non‐cardiomyocyte‐localized Cx43 was mostly localized in cardiac fibroblasts, but not in monocyte or capillary endothelial cells (Figure S1E, S2A, and S3A).

**Figure 2:**
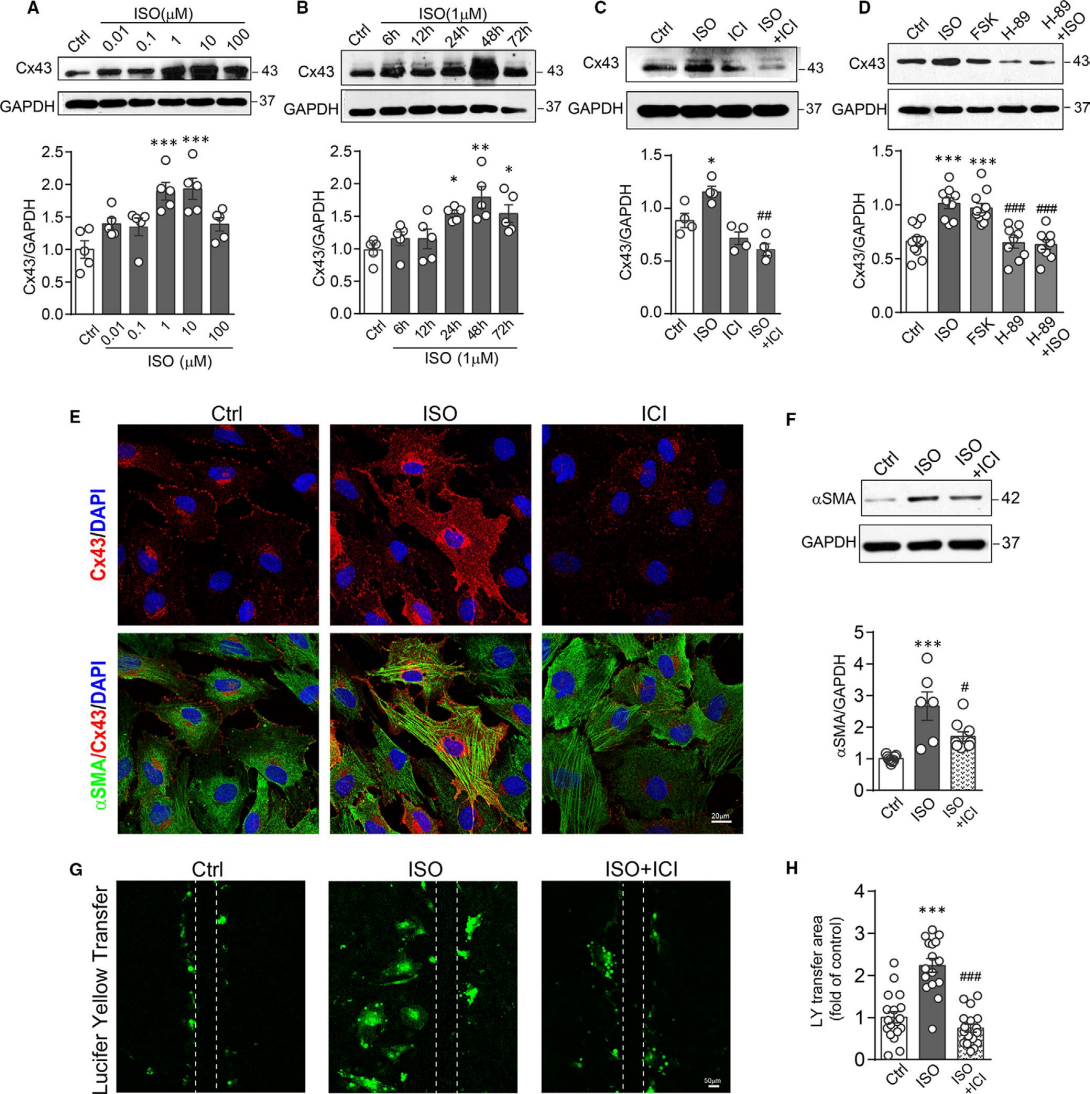
ISO treatment of cultured cardiac fibroblasts enhanced Cx43 expression and intercellular coupling through β_2_‐AR/cAMP/PKA pathway. Western blotting images and quantification of band intensity for Cx43 expression in fibroblasts (A) treated with ISO at different concentrations (0.01 ~ 100 μmol/L) for 24 h (n = 5 independent isolation/5 hearts), or (B) treated with ISO (1 μmol/L) for various durations (6 ~ 72 h, n = 5 independent isolation/5 hearts), or (C) treated with ISO (1 μmol/L), ICI (0.1 μmol/L), ICI plus ISO for 24 h (n = 5 independent isolation/5 hearts), and (D) treated with ISO, forskolin (100 μmol/L), H‐89 (0.1 μmol/L) and H89 plus ISO for 24 h (n = 9 independent isolation/5 hearts). E, Representative fluorescence images of Cx43 (red) and α‐SMA (green) in fibroblasts treated with ISO, ICI plus ISO for 48 h. Scale bar: 20 µm. F, Western blotting images and quantification of band intensity for α‐SMA expression in fibroblasts treated with ISO or ICI plus ISO for 48 h (n = 5 independent isolation/5 hearts). G, Representative fluorescence images and (H) quantification of Lucifer yellow transfer area after scrape‐loading by dye transfer assay in fibroblasts treated without or with ISO and ISO plus ICI for 48 h. (n = 15 assays from 5 independent isolation/5 hearts). Scale bar: 50 µm. Data are expressed as mean ± SEM. *P < .05, **P < .01 and ***P < .001 vs control, ^#^ *P* < .05, ^##^ *P* < .01 and ^###^ *P* < .001 vs ISO. Statistical significance was determined by one‐way ANOVA followed by Tukey's multiple comparisons test

To further determine whether fibroblasts respond to ISO with up‐regulation of Cx43, we performed experiments using cultured adult mouse cardiac fibroblasts. Fibroblasts stimulated with ISO showed up‐regulated Cx43 expression in a concentration‐ and time‐dependent manner (Figure 2A and B). Concomitant addition of ICI‐118551 (0.1 μmol/L) abolished ISO‐induced Cx43 up‐regulation (Figure 2C), the finding in consistent with the notion that fibroblasts are equipped only with β_2_‐AR. We then tested in fibroblasts effects of the adenylate cyclase activator forskolin (100 μmol/L, 24 hours) or PKA inhibitor H‐89 (0.1 μmol/L, 24 hours). Whilst Cx43 expression was increased by 46% with forskolin, ISO‐stimulated Cx43 expression was abolished by H‐89 (Figure 2D). Collectively, these in vitro results suggest that elevated Cx43 expression in fibroblasts by ISO was mediated through the β_2_‐AR/cAMP/PKA pathway.

Furthermore, ISO treatment of fibroblasts in vitro markedly up‐regulated the expression of α‐SMA, a marker of myofibroblasts. This effect of ISO was also reversed by administration of ICI (Figure 2E and F). Cx43 expression and distribution were determined by immunofluorescence staining. As shown in Figure 2E, ISO‐stimulated fibroblasts exhibited enhanced immunoreactive punctate signals that localized at the interface with adjacent cells as well as around nuclear. Meanwhile, the prominent α‐SMA‐positive microfilament bundles were formed in ISO‐treated cells which were reduced by addition with ICI.

Next, we examined whether up‐regulation of Cx43 by ISO in fibroblasts influenced intercellular coupling. Because gap junction channels composed of Cx43 are highly permeable to the fluorescent dye Lucifer yellow,^34^ we employed the scrape‐loading dye transfer assay in cultured fibroblasts to assess gap junction activity. The Lucifer yellow transfer area in ISO‐stimulated fibroblasts was 2.6‐fold higher than that of control cells, which once again was reversed by ICI (Figure 2G and H).

### 3.3 Cardiomyocyte β_2_‐AR overexpression caused Cx43 up‐regulation and lateralization

**Figure 3:**
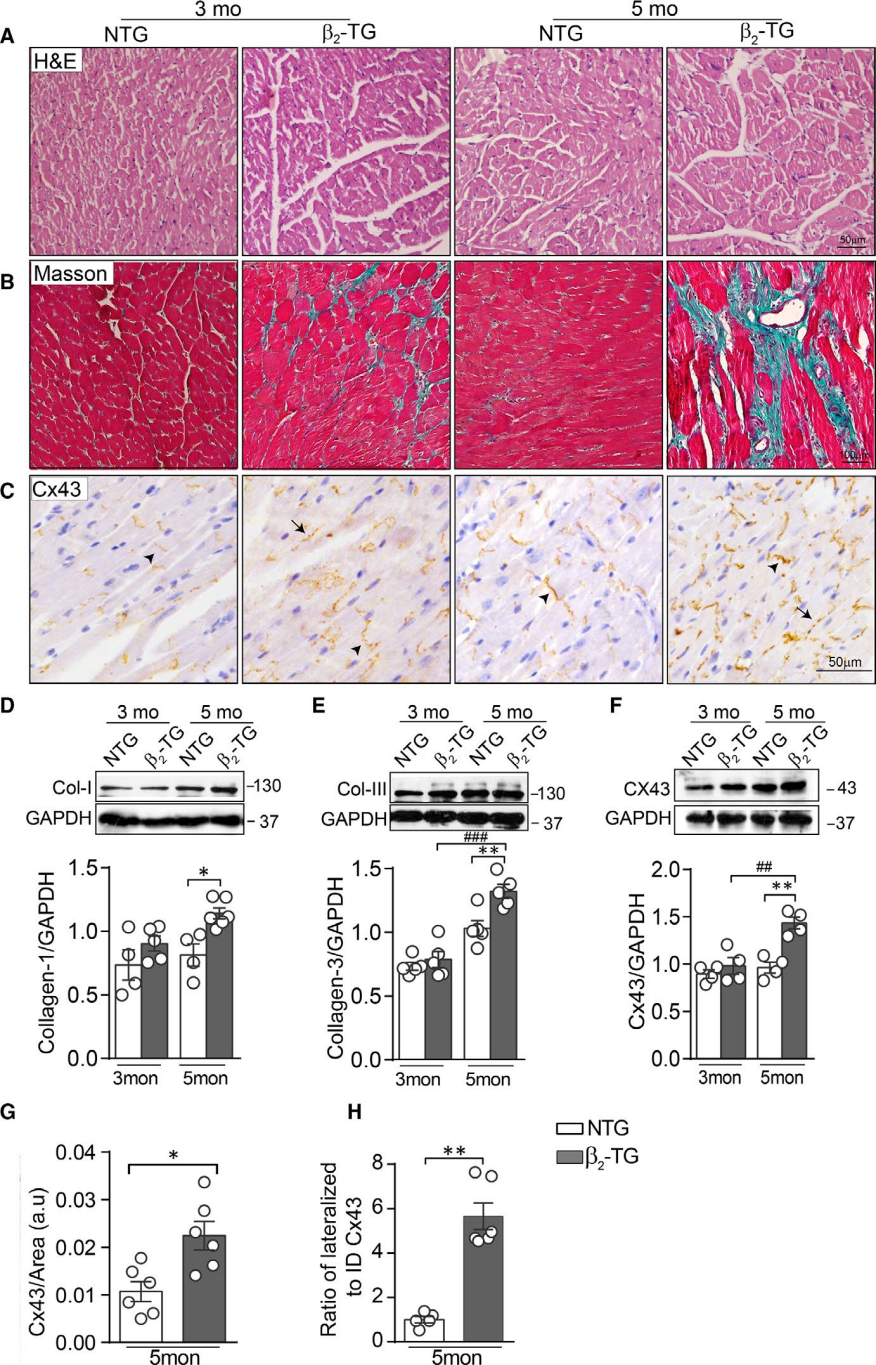
β_2_‐AR overexpression in cardiomyocytes led to age‐related fibrosis with altered Cx43 expression and localization. Representative images of HE staining (A), Masson's trichrome staining (B) and (C) Cx43 IHC staining in LV sections from NTG and β_2_‐TG mice aged at 3 and 5 months (Scale bar: 50 µm in A and C, 100 µm in B). Arrows indicate lateralized Cx43 and arrowheads for ID Cx43 localization. Western blotting images and quantification of band intensity for expression of (D) Col‐I, (E) Col‐III and (F) Cx43 in LV tissue lysate (n = 5 mice/group). Quantificational analysis for (G) total Cx43 positive area (n = 5 mice/group) and (H) the ratio of lateral to ID localized Cx43 (n = 5 mice/group) in LV sections of 5‐month‐old NTG and β_2_‐TG mice. Data were expressed as mean ± SEM. *P < .05, **P < .01 and ***P < .001 vs NTG; ^##^ *P* < .01 and ^###^ *P* < .001 vs β_2_‐TG mice at 3 months. Statistical significance was determined by one‐way ANOVA followed by Tukey's multiple comparisons test (D‐F) or two‐tailed unpaired Student's *t* test (G‐H)

In the diseased myocardium, β_2_‐ARs are redistributed across cardiomyocyte membrane thereby mediating from highly compartmentalized cAMP signalling to diffuse and propagating signalling.^7^ Hence, we used cardiomyocyte‐restricted overexpression of β_2_‐AR (β_2_‐TG) mouse model to assess the effect of chronic β_2_‐AR activation on the expression and distribution of Cx43. In consistent with previous reports, β_2_‐TG mice developed age‐dependent pathological hypertrophy (data not shown) and fibrosis, evident by histology of LV sections (Figure 3A and B) and elevated expressions of Col‐I and Col‐III by Western blotting (Figure 3D and E). These results indicated that similar to the ISO model, cardiomyocyte‐restricted β_2_‐AR activation leads to myocardial remodelling notably fibrosis.

We then examined changes in expression and localization of Cx43 in the LV myocardium of 5‐month‐old β_2_‐TG mice. Immunohistochemistry staining of the LV of β_2_‐TG mice exhibited significant increase in Cx43‐positive staining, with increased lateralization whilst reduced ID localization (Figure 3C, G, and H). In consistent with this histological finding, Western blotting revealed a 50% increase in protein level of Cx43 in β_2_‐TG vs NTG hearts (Figure 3F). Similar to ISO‐treated group, immunofluorescence staining revealed that Cx43 in non‐cardiomyocytes was mostly localized in cardiac fibroblasts in 5‐month‐old β_2_‐TG mice (Figure S1E, S2B and S3B).

### 3.4 β_2_‐AR activation suppressed Cx43 expression and localization in cardiomyocytes

To further examine changes in the expression and localization of Cx43 in cardiomyocytes, we prepared cardiomyocytes from adult mice or β_2_‐TG mice for immunofluorescence staining.

**Figure 4:**
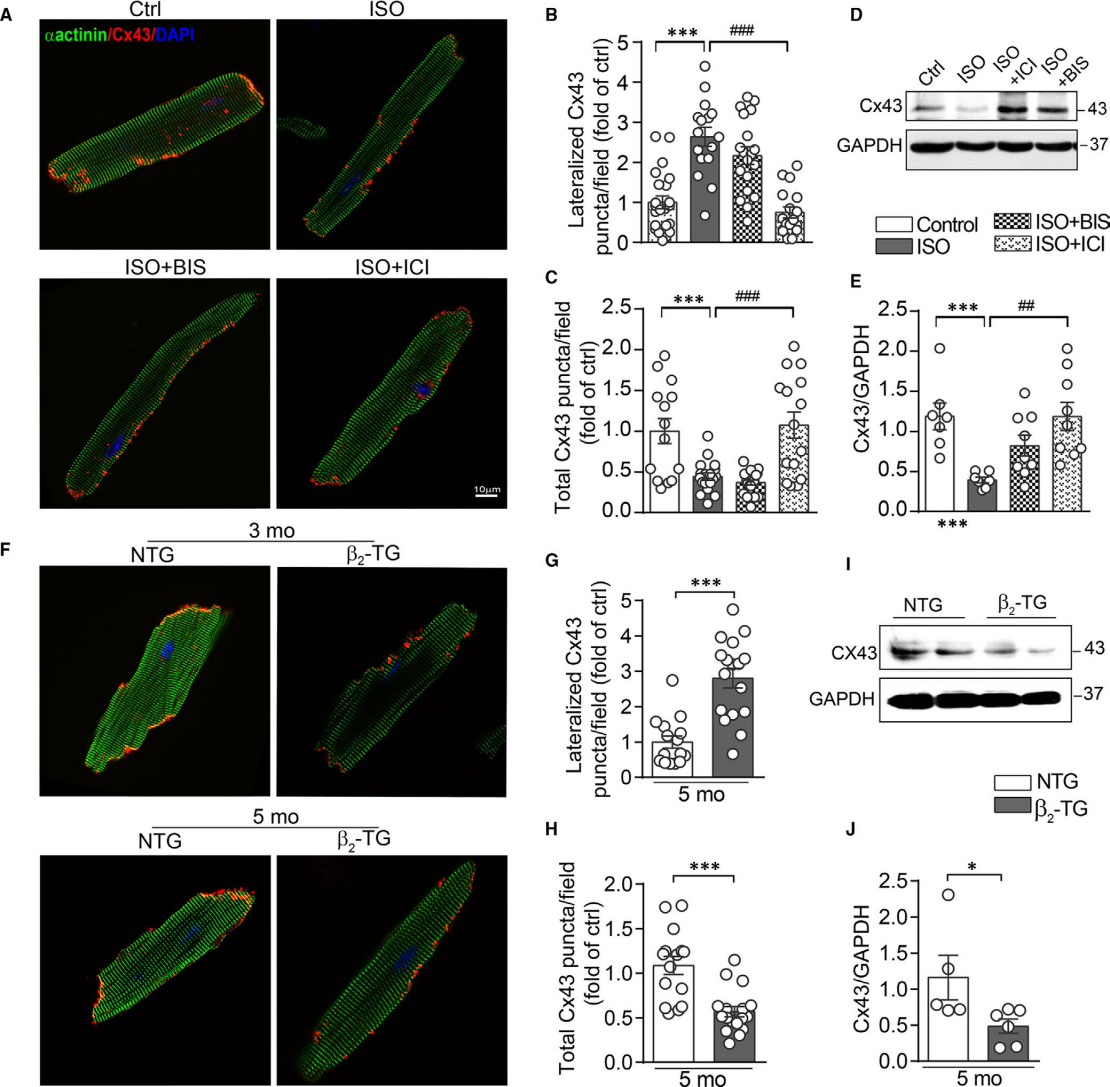
Activation of β_2_‐AR suppressed Cx43 expression and localization in primary adult mouse cardiomyocytes. A, Representative fluorescence images for Cx43 (red) and α‐actinin (green) in cardiomyocytes treated with ISO (1 μmol/L), ISO plus BIS (0.1 μmol/L) or ICI (0.1 μmol/L) for 48 h. Scale bar: 10 µm. Quantitative analysis for (B) the lateralized Cx43 area per image field, (C) the total Cx43‐positive area (include ID localized Cx43) in cardiomyocytes (n = 16‐22 cells/group from 5 hearts). D and E, Western blotting images and quantification of band intensity for Cx43 expression in cardiomyocytes treated with ISO, ISO plus BIS or ICI for 48 h (n = 5 independent isolation/2 hearts). F, Representative fluorescence images for Cx43 (red) and α‐actinin (green) in cardiomyocytes isolated from NTG and β_2_‐TG mice at 3 and 5 months of age. Quantificational analysis for (G) the lateralized Cx43 fluorescence puncta area per image field, (H) the total Cx43‐positive area in cardiomyocytes isolated from 5‐month‐old NTG and β_2_‐TG mice (n = 15~17 cells/group from 5 hearts). I and J, Western blotting images and quantification of band intensity for Cx43 expression in cardiomyocytes isolated from 5‐month‐old NTG and β_2_‐TG mice (n = 5 independent isolation/2 hearts). Data were expressed as mean ± SEM. *P < .05 and ***P < .001 vs Ctrl or NTG; ^##^ *P* < .01 and ^###^ *P* < .001 vs ISO. Statistical significance was determined by one‐way ANOVA followed by Tukey's multiple comparisons test (B‐E) or two‐tailed unpaired Student's *t* test (G‐J)

In primary cardiomyocytes, 48‐h ISO treatment induced an increase in lateralized Cx43‐positive puncta, whilst total Cx43‐positive puncta were reduced (Figure 4A‐C). Western blotting further verified the reduced expression of total Cx43 in ISO‐treated cardiomyocytes (Figure 4D and E). These changes by ISO in cardiomyocytes were abolished by ICI‐118551 but not by bisoprolol (Figure 4A‐E).

Similar to ISO‐treated cardiomyocytes, in cardiomyocytes isolated from 5‐month‐old β_2_‐TG hearts, lateralized Cx43‐positive puncta were significantly high whilst the total Cx43 expression level, estimated by both puncta and by Western blotting, was lower relative to NTG cardiomyocytes (Figure 4F‐J).

### 3.5 β‐AR activation in cardiomyocytes evoked IL‐18 secretion contributing to Cx43 up‐regulation in fibroblasts *via* a paracrine mechanism

**Figure 5:**
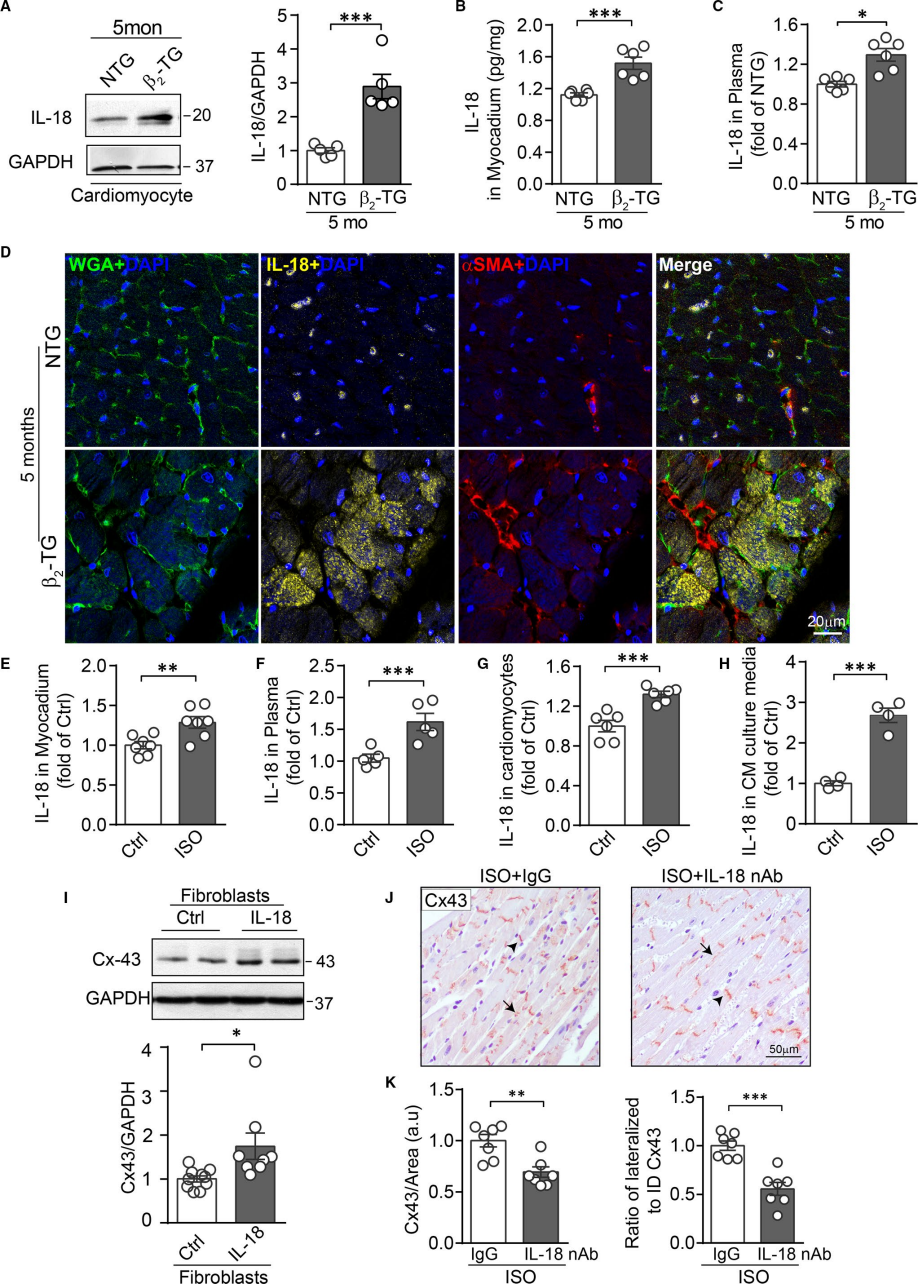
Cardiomyopcyte‐derived IL‐18 by β‐AR activation contributed to Cx43 up‐regulation in fibroblasts in a paracrine fashion. Quantification of IL‐18 expression in cardiomyocytes by Western blotting analysis (A) (n = 6 mice/group), or concentrations by ELISA (B) in myocardium tissue lysate (n = 6 mice/group) and (C) in plasma (n = 6 mice/group) in 5‐month‐old NTG and β_2_‐TG mice. D, Immunofluorescence staining of WGA (green), IL‐18 (yellow), α‐SMA (red) and DAPI (blue) in LV myocardium from 5‐month‐old NTG and β_2_‐TG mice. Scale bar: 20 µm. Concentration of IL‐18 (E) in myocardium tissue lysate and (F) in plasma from control and 7‐day after ISO treatment measured by ELISA (n = 5‐7 mice/group). Concentration of IL‐18 (G) in primary cardiomyocytes in culture or (H) culture media harvested from cardiomyocytes with ISO treatment (1 μmol/L, 48 h) by ELISA (n = 4‐6 independent isolation/2 hearts). I, Western blotting images and quantification of band intensity for Cx43 expression in adult mouse cardiac fibroblasts treated with IL‐18 (10 ng/mL) for 48 h (n = 5 independent isolation/5 hearts). J, Representative images of Cx43 IHC staining in LV sections and (K) quantificational analysis for Cx43 positive area and the ratio of lateral to ID localized Cx43 area in LV sections of mice treated daily with IL‐18 nAb or IgG for 7 d commencing from ISO infusion (n = 7 mice/group). Scale bar: 50 µm. Arrows indicate lateralized Cx43 and arrowheads for ID Cx43 localization. Data were expressed as mean ± SEM. *P < .05, **P < .01 and ***P < .001 vs NTG or control. Statistical significance was determined by two‐tailed unpaired Student's *t* test

Previous study showed that upon β‐AR stimulation, IL‐18 is a crucial contributor in cardiac pathological remodelling.^35^ To explore whether IL‐18 was responsible for the alterations of Cx43 seen in both β_2_‐TG and ISO models, the expression level of IL‐18 in the heart was determined. In cardiomyocytes freshly isolated from 5‐month‐old β_2_‐TG mice, Western blotting analysis revealed approximately 3‐fold increase in IL‐18 expression relative to NTG cells (Figure 5A). Consistently, ELISA assay detected a 30% increase of IL‐18 level in the LV homogenate lysate as well as in the plasma from β_2_‐TG vs NTG mice (Figure 5B and C). Furthermore, triple immunofluorescence staining revealed cardiomyocyte localization of IL‐18, but not co‐localized with fibroblast marker α‐SMA, suggesting that in β_2_‐TG heart cardiomyocytes were the main cell type producing and releasing IL‐18 (Figure 5D). Consistently, increased IL‐18 levels were detected in the myocardial homogenate lysate or plasma of ISO‐treated mice (Figure 5E and F). Furthermore, in primary cultured cardiomyocytes, ISO treatment also increased IL‐18 levels in both cardiomyocyte and the culture media (Figure 5G and H). These results suggest a constant release of IL‐18 by cardiomyocytes upon β‐AR activation.

We further tested the potential that IL‐18 released by cardiomyocytes upon β_2_‐AR activation mediates Cx43 up‐regulation in fibroblast. Mouse cardiac fibroblasts were stimulated with recombined IL‐18 (10 ng/mL) for 48 hours. Western blotting detected Cx43 up‐regulation by IL‐18 stimulation (Figure 5I). Moreover, in vivo inhibition of IL‐18 was achieved by daily injection with IL‐18 neutralizing antibody (IL‐18 nAb) for 7 consecutive days commencing with ISO infusion. By the Cx43 immunohistochemical staining in LV sections, the increment of total Cx43 expression level and lateral redistribution in the LV myocardium were significantly reduced by IL‐18 nAb compared to the IgG treated group (Figure 5J and K).

## 4 DISCUSSION

**Figure 6:**
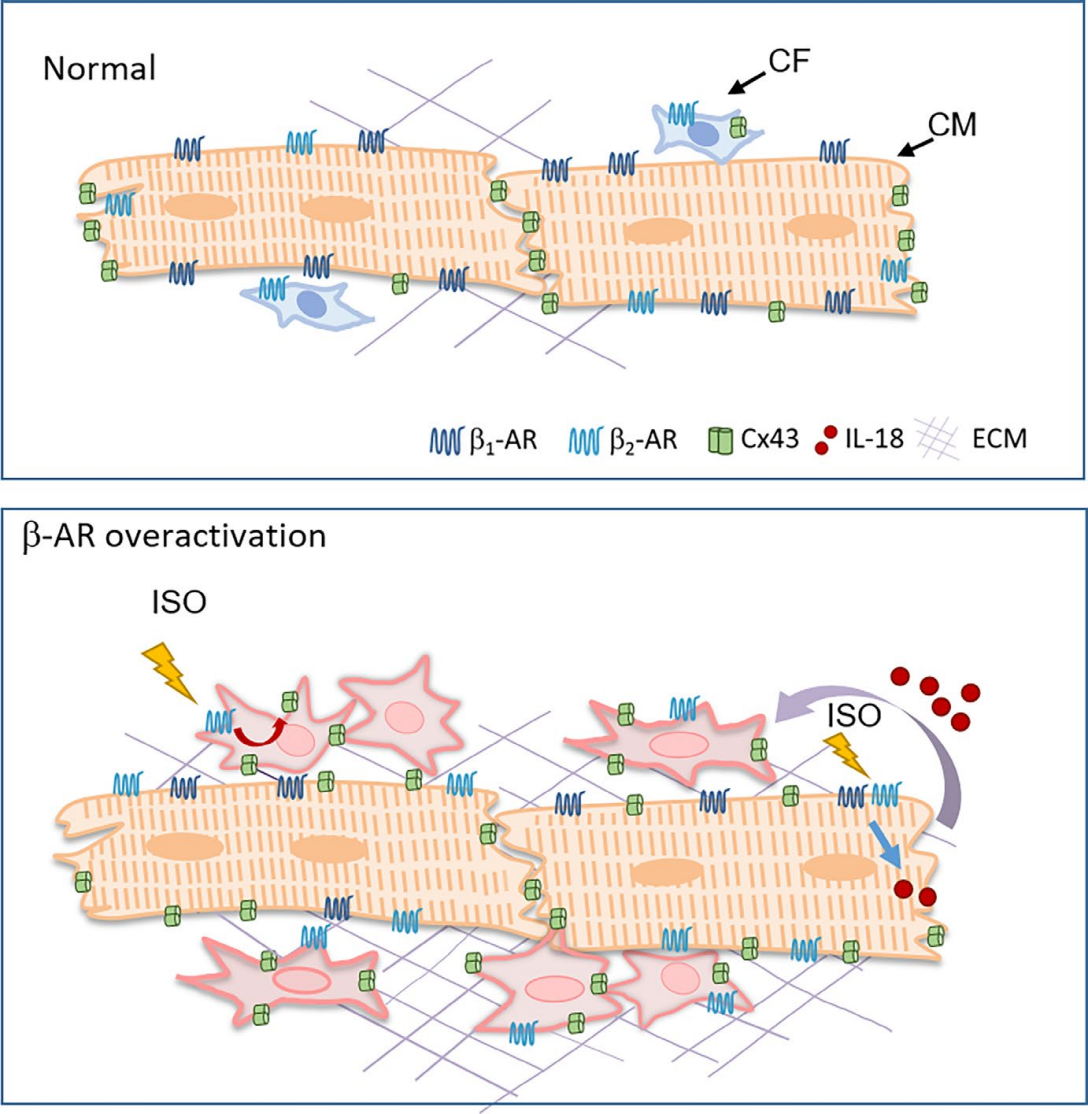
Schematics depicting gap junction remodelling in cardiomyocytes and fibroblasts induced by cardiac β‐AR activation. In both ISO stimulation and β_2_‐TG mouse models, β‐AR activation in cardiomyocytes suppressed Cx43 expression and shifted Cx43 localization from ID to the lateral side of cardiomyocytes. Meanwhile, Cx43 expression in fibroblasts, when tested in vivo and in vitro, were up‐regulated *via* direct activation of β_2_‐AR/cAMP/PKA signalling cascade as well as stimulation by IL‐18 released from cardiomyocytes upon β‐AR activation (curved arrow). These changes would increase the probability of intercellular coupling via gap junctions

Despite numerous reports documenting gap junction remodelling in fibrotic cardiomyopathy, the underlying molecular and cellular mechanisms are unknown.^36^ In the present study, two novel findings have been made. Firstly, in both ISO‐stimulated and genetic β_2_‐TG mouse models, β‐AR activation in cardiomyocytes suppressed Cx43 expression, which was associated with an increased lateral vs ID ratio of Cx43, suggesting redistribution of gap junctions in cardiomyocytes (Figure 6). Secondly, in both models Cx43 expression in fibroblasts was up‐regulated by β‐AR stimulation directly through activation of the β_2_‐AR/cAMP/PKA signalling and indirectly through IL‐18 released from cardiomyocytes (Figure 6).

Cx43 is the most abundant subunit of gap junction in ventricular cardiomyocytes as well as in fibroblasts and inflammatory cells.^19^ Using cardiac biopsies from patients with hypertrophic, dilated or ischaemic cardiomyopathies, studies have revealed an increased in early stage but suppressed overall expression of Cx43 in the end‐stage of cardiomyopathy.^17^, ^33^ The present study extends these findings by showing cellular characteristics of Cx43 distribution in ISO‐treated or β_2_‐TG mouse hearts with signs of early decompensation. Studies in vitro have implicated fibroblast‐cardiomyocyte coupling through gap junctions that bears pro‐arrhythmic potential albeit in vivo evidence is still missing.^37^ In the present study, we found that enhanced β_2_‐adrenergic receptor activity preferably stimulates expression of Cx43 in fibroblasts and that, whilst expression level was reduced in cardiomyocytes, increased proportion of Cx43 was laterally localized. Thus, these changes would be expected to increase Cx43 density at cardiomyocyte‐fibroblast interface with increased probability of hetero‐cellular coupling, which may contribute to the spontaneous ectopic automaticity, re‐entry and triggered after depolarizations.^38^, ^39^ Indeed, spontaneous ventricular arrhythmias have been reported in β_1_‐TG and β_2_‐TG mice.^4^, ^6^ Thus, our findings suggest that enhanced β‐AR activity is a potential pre‐requisite for Cx43‐mediated cardiomyocyte‐fibroblast electronic coupling in vivo.

Cardiomyocytes are equipped with both β_1_‐ and β_2_‐AR with distinct features of G‐protein coupling and intracellular signalling.^40^ Whilst both β_1_‐ and β_2_‐AR subtypes use Gs‐cAMP‐PKA signalling to mediate positive chronotropy, inotropy and lusitrop, previous studies in human ventricular myocytes revealed that the coupling between Gs and β_2_‐AR are tighter than β_1_‐AR.^41^, ^42^ Besides, β_2_‐AR also couples with inhibitory Gi‐protein, which can blunt β_1_‐AR signalling in healthy and diseased myocardium.^43^, ^44^, ^45^ In neonatal rat cardiomyocytes, acute activation of β_2_‐AR, but not β_1_‐AR, signalling regulated the phosphorylation of Cx43.^46^ In this study, our results also indicated that suppressed expression and redistribution of Cx43 both in *vivo* and in primary cultured cardiomyocytes were mainly mediated through β_2_‐AR, whilst the contribution by β_1_‐AR was moderate. Further work is required to determine the distinct regulations of Cx43 by β_1_‐ and β_2_‐AR.

β‐AR signalling in fibroblasts in relation to Cx43 expression remains unexplored. Whilst contributing to over 80% of total myocardial mass, cardiomyocytes only account for less than 25% of all cell numbers,^47^ and fibroblasts form a major cell population, particularly in the diseased heart, contributing to pathological remodelling.^48^ In the present study, we further explored Cx43 expression in fibroblasts and inflammation cells in response to β‐AR stimulation. Our data revealed that activation of β_2_‐AR, but not β_1_‐AR, induced Cx43 up‐regulation in fibroblasts, the finding in keeping with the notion that only β_2_‐AR exists in fibroblasts.^14^ β_2_‐AR is known to couple to different G proteins and diverse downstream signalling pathways,^49^ and our results from fibroblasts indicate that it is the β_2_‐AR/cAMP/PKA signalling pathway that mediates the change in Cx43 expression. Emerging evidence suggests that inter‐fibroblast coupling by gap junctions promotes fibroblast activation.^50^ Our data indicated that β_2_‐AR mediated Cx43 up‐regulation increases inter‐fibroblast coupling via gap junctions and that Cx43 up‐regulation was accompanied with increased α‐SMA expression, indicating that Cx43 acts as an activator for α‐SMA synthesis. Such mechanism has been indicated by a few in vitro studies on pulmonary or cardiac fibroblasts stimulated with angiotensin II or TGF‐β.^32^, ^50^ Previous studies have also revealed inflammatory infiltration of the myocardium in both ISO and β_2_‐TG models.^51^, ^52^ By immunohistochemical staining, whilst monocytes were present in the myocardium in both models, very few monocytes were co‐localized with Cx43, suggesting that myocardial monocytes contribute little to the overall level of Cx43 in the setting of β‐AR activation.

Interestingly, in the β_2_‐TG model, the β_2_‐AR genetic targeting was confined to cardiomyocytes and yet severe fibrosis was evident, suggesting that overactivation of β_2_‐AR in cardiomyocytes act in a paracrine manner leading to both fibrosis and Cx43 up‐regulation in fibroblasts. Enhanced intercellular coupling by gap junction would be in favour of fibroblast activation and proliferation.^50^ Cardiac β‐Adrenergic receptor stimulation is sufficient to induce expression of several pro‐inflammatory cytokines, including tumour necrosis factor (TNF)‐α, IL‐1β, IL‐6 and IL‐18.^35^ IL‐18 cleavage in cardiomyocytes resulted in secretion of IL‐18‐dependent cytokines, which, in turn, triggers macrophage infiltration and pathological remodelling.^53^ Our data further revealed that acute or chronic activation of β_2_‐AR in cardiomyocytes promotes production and release of IL‐18, which acts in paracrine fashion in activating Cx43 expression in fibroblasts (Figure 6). Indeed, IL‐18 neutralization in vivo inhibited both Cx43 up‐regulation and redistribution after ISO stimulation. These findings indicate a novel signal coupling between cardiomyocytes and fibroblasts that is responsible at least, in part, for Cx43 remodelling in fibrotic cardiomyopathy. Further study is warranted to illustrate the potential contribution of this mechanism in arrhythmogenesis associated with enhanced sympatho‐β‐adrenergic receptors activity in the heart.

## 5 CONCLUSIONS

Our findings suggest that activation of β‐adrenergic receptors system attenuates cardiomyocyte expression of Cx43 together with redistribution. Up‐regulated Cx43 expression in fibroblasts is achieved directly by β_2_‐AR and indirectly through a paracrine signalling that involves cardiomyocyte‐derived IL‐18 (Figure 6). It is postulated that the suppression of β_2_‐AR signalling forms an attractive approach in combating arrhythmias and cardiac fibrosis.

## CONFLICT OF INTEREST

None declared.

## AUTHOR CONTRIBUTION

**Yi Zhang:** Conceptualization (equal); Data curation (lead); Formal analysis (lead); Funding acquisition (equal); Investigation (lead); Writing‐original draft (equal); Writing‐review & editing (equal). **Meng‐Chen Hou:** Data curation (equal); Formal analysis (equal); Investigation (equal); Validation (equal). **Jing‐Jing Li:** Data curation (supporting); Formal analysis (supporting); Methodology (supporting); Validation (supporting); Visualization (supporting). **Ying Qi:** Data curation (supporting); Formal analysis (supporting). **Yu Zhang:** Data curation (supporting); Formal analysis (supporting). **Gang She:** Data curation (supporting); Formal analysis (supporting). **Yu‐Jie Ren:** Data curation (supporting); Formal analysis (supporting). **Wei Wu:** Data curation (supporting); Formal analysis (supporting). **Zheng‐Da Pang:** Data curation (supporting); Formal analysis (supporting); Resources (supporting); Visualization (supporting). **Wenjun Xie:** Funding acquisition (supporting); Writing‐original draft (supporting); Writing‐review & editing (supporting). **Xiu‐Ling Deng:** Conceptualization (lead); Funding acquisition (lead); Project administration (lead); Resources (lead); Supervision (lead); Writing‐original draft (lead); Writing‐review & editing (lead). **Xiao‐Jun Du:** Conceptualization (equal); Funding acquisition (equal); Project administration (lead); Supervision (lead); Writing‐original draft (lead); Writing‐review & editing (lead).

## Supporting information

Supplementary Material

## Data Availability

The data used to support the findings of this study are available from the corresponding author upon request.
