# Supplementary material for: Cardiac β‐adrenergic receptor activation mediates distinct and cell type‐dependent changes in the expression and distribution of connexin 43
Source: J Cell Mol Med. 2020 Jun 24;24(15):8505–17. doi: 10.1111/jcmm.15469 (PMC7412418; doi:10.1111/jcmm.15469)
Supplement: Supplementary file 1 — Supplementary Material [file JCMM-24-8505-s001.doc]

**Supplemental data**

**Cardiac-adrenergic receptor activation mediates distinct and cell type-dependent changes in the expression and distribution of connexin 43**

**Short title:** Myocardial gap junction remodeling and β-AR activation

Yi Zhang^1#^, Meng-Chen Hou^1, 2#^, Jing-Jing Li^3^, Ying Qi^3^, Yu Zhang^1^, Gang She^1^, Yu-Jie Ren^2^, Wei Wu^1^，Zheng-Da Pang^1^, Wenjun Xie^3^, Xiu-Ling Deng^1, 4*^, Xiao-Jun Du^1, 5*^

^1^Department of Physiology and Pathophysiology, School of Basic Medical Sciences, Xi’an Jiaotong University Health Science Center, Xi’an, Shaanxi, China

^2^Department of Pathology, Xi’an Guangren Hospital, Xi’an Jiaotong University Health Science Center, Xi’an, Shaanxi, China

^3^The Key Laboratory of Biomedical Information Engineering of Ministry of Education, School of Life Sciences and Technology, Xi’an Jiaotong University, Xi'an, Shaanxi, China

^4^Key Laboratory of Environment and Genes Related to Diseases, Ministry of Education, Xi’an Jiaotong University Health Science Center, 710061, Shaanxi, China

^5^Experimental Cardiology Laboratory, Baker Heart and Diabetes Institute, Melbourne, Australia

**
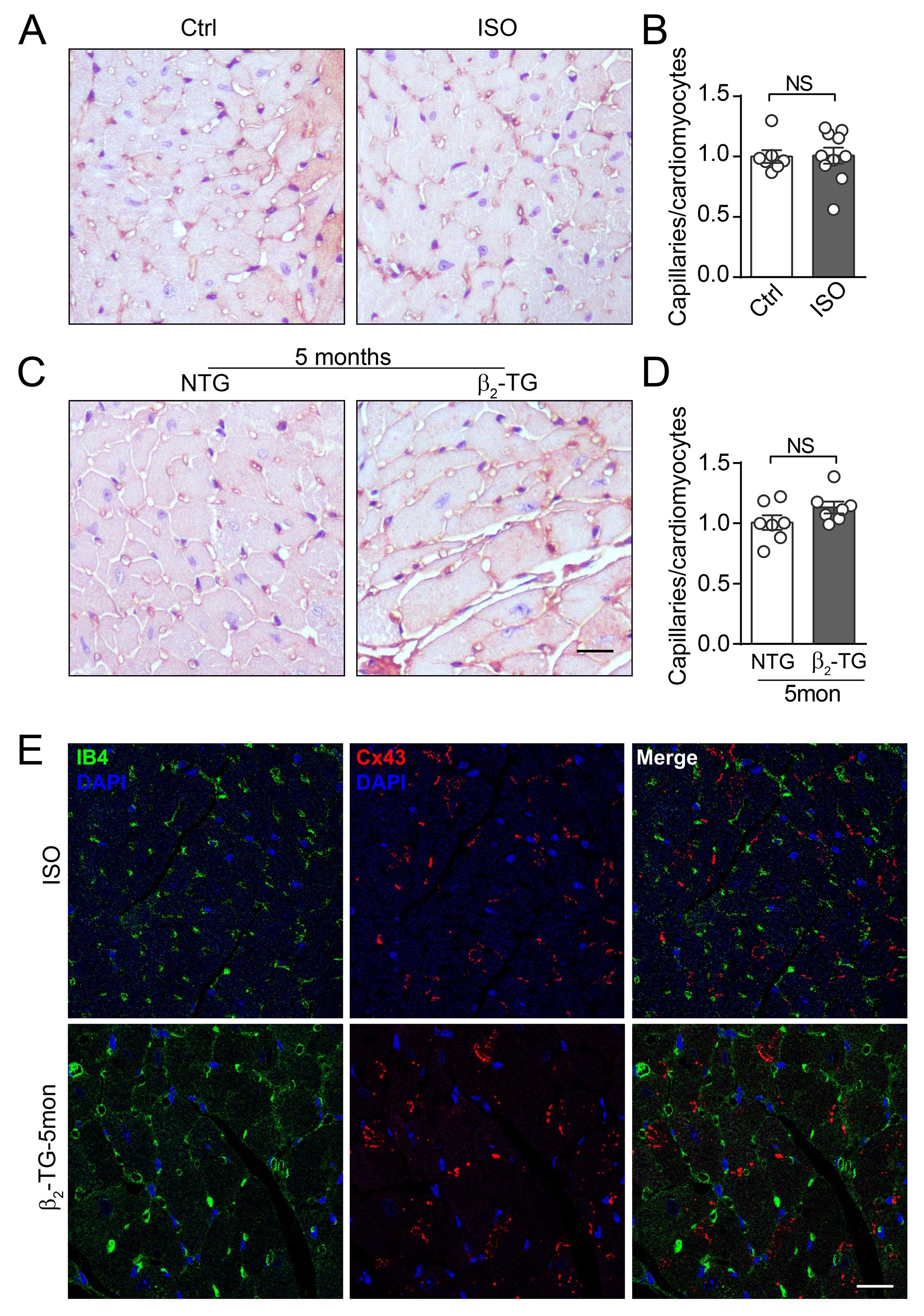
**

**Supplemental Figure 1. Cardiac activation of β-AR affects neither capillary density nor co-localization of Cx43 with endothelial cells in LV myocardium from ISO-treated or 5-month-old β_2_-TG mice.** Representative images of VE-Cadherin IHC staining of coronary capillaries and quantification of capillary density in LV myocardium from control and 7-day ISO-treated group **(A, B)** and from 5-month-old NTG and β_2_-TG mice **(C, D)**. Capillary density was quantified as the number of capillaries per cardiomyocyte in each heart. (n=7 mice/group). **(E)** Immunofluorescence staining of Isolectin B4 (IB4) for endothelial cells (green), Cx43 (red) and DAPI (blue) in LV myocardium from 7-day ISO-treated group (upper panel) and 5-month-old β_2_-TG mice (lower panel). Scale bar: 20 µm. NS: no significant.

**
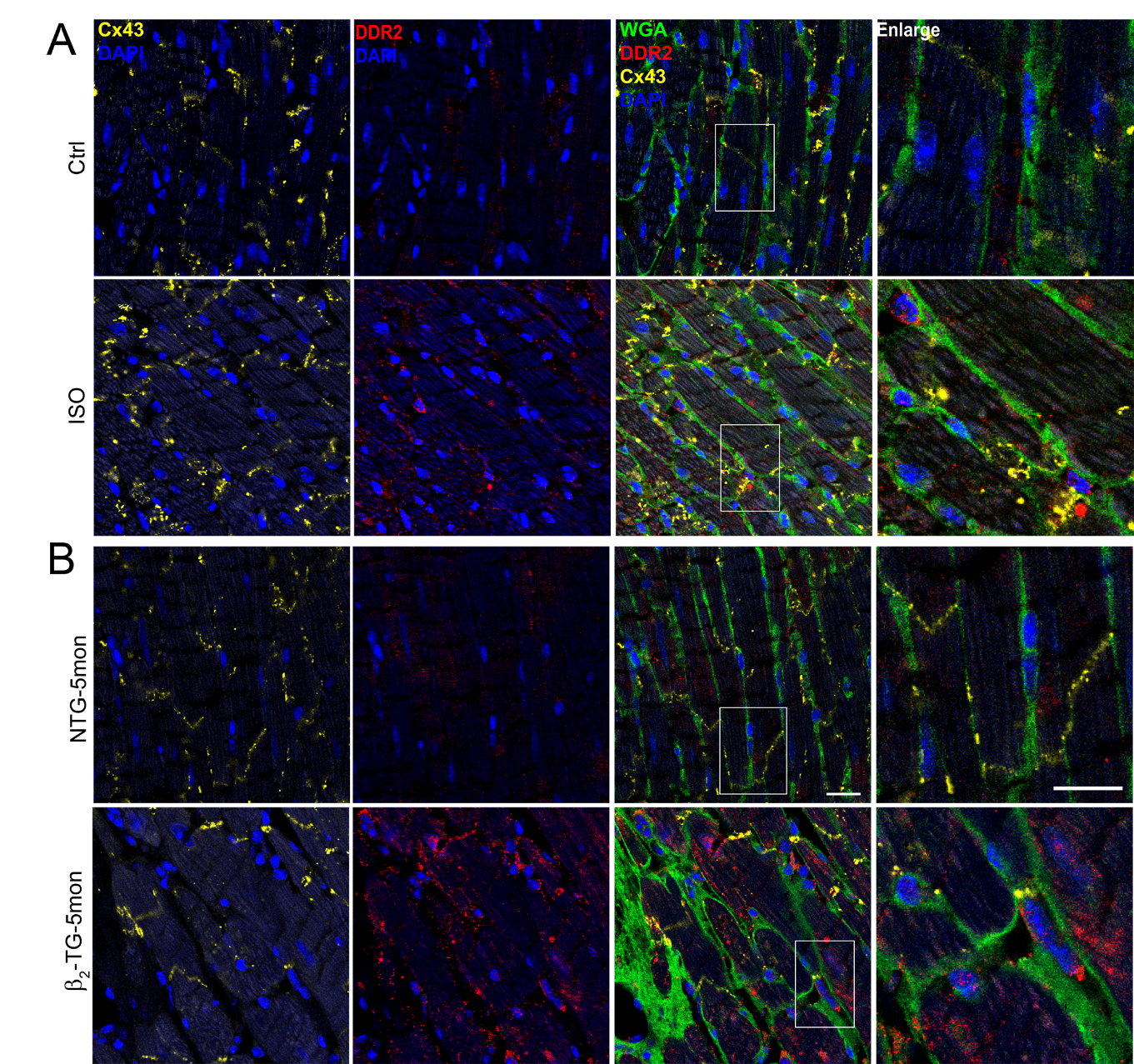
**

**Supplemental Figure 2. β-AR stimulation promoted Cx43 expression in fibroblasts with increased localization at the cardiomyocyte-fibroblast interface.** Immunofluorescence staining of WGA (green), DDR2 (red), Cx43 (yellow) and DAPI (blue) in LV myocardium from 5-month-old NTG and β_2_-TG mice **(A)** or wild-type mice received 7-day treatment with saline or ISO **(B)**. Scale bar: 20 µm. White boxed areas were amplified views enlarged in the right panels. Scale bar: 20 µm.

**
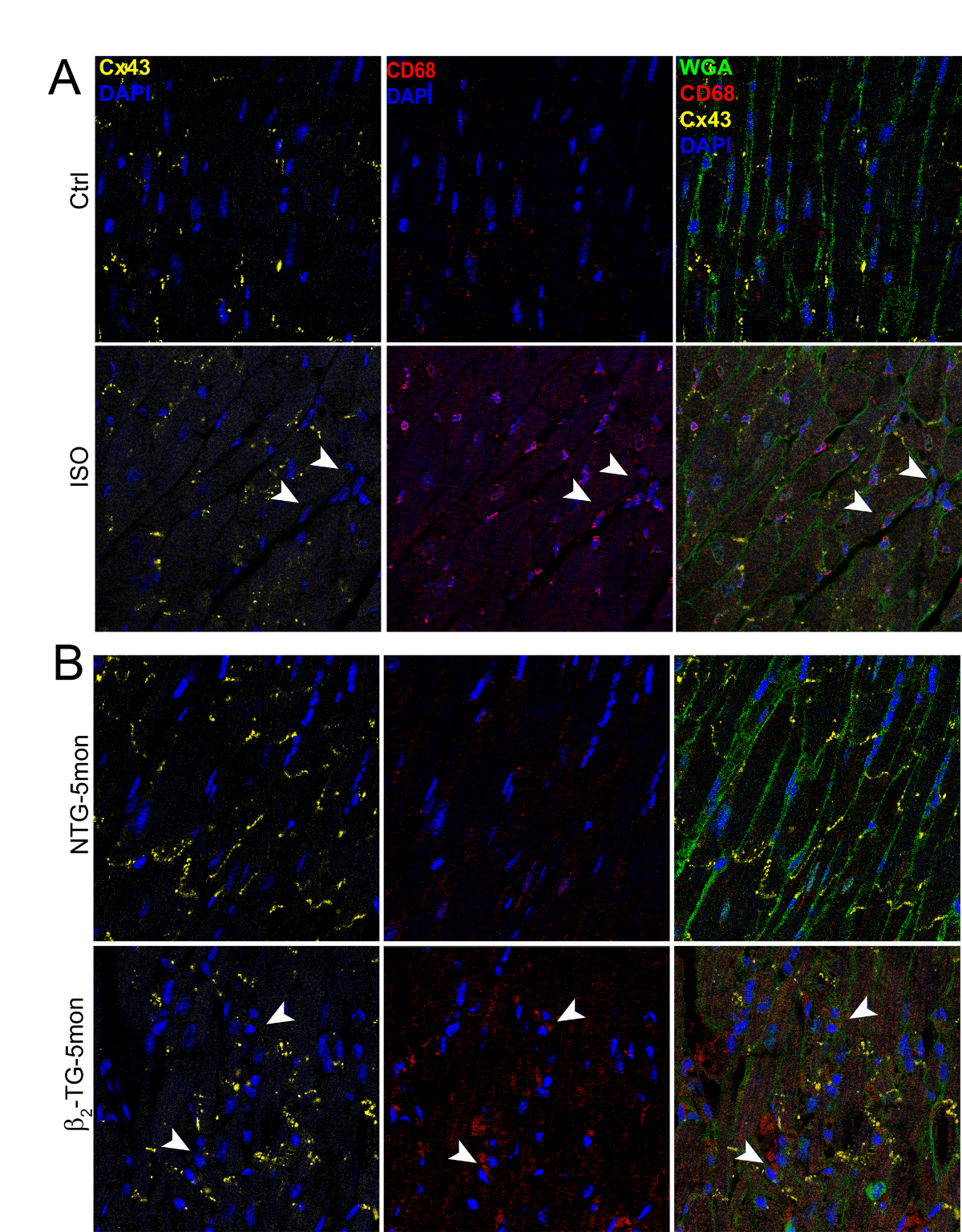
**

**Supplemental Figure 3. Few Cx43 co-localized with monocytes in the myocardium of hearts from ISO-treated mice or β_2_-TG mice.** Immunofluorescence staining of WGA (green), CD68 (red), Cx43 (yellow) and DAPI (blue) in left ventricular myocardium from control and 7-day ISO treated groups **(A)** or from 5-month-old NTG and β_2_-TG mice **(B)**. White arrowheads indicate CD68 positive staining. Scale bar: 20 µm.

**Supplemental Table 1.** Organ weights and echocardiographic measures in mice with and without treatment of ISO for 7 days.

|  | Sham (n=10) | ISO (n=8) | P-value |  |
| --- | --- | --- | --- | --- |
| **Organ weights** |  |  |  |  |
| Body weight, g | 27.6±4.4 | 28.2±3.3 | NS |  |
| LV/TL, mg/mm | 5.32±0.56 | 5.89±0.27 | <0.05 |  |
| RV/TL, mg/mm | 1.24±0.18 | 1.49±0.07 | <0.01 |  |
| Atria/TL, mg/mm | 0.61±0.05 | 0.80±0.16 | <0.01 |  |
| Heart/TL, mg/mm | 7.11±0.76 | 7.98±0.69 | <0.01 |  |
| Lungs/TL, mg/mm | 8.70±0.36 | 9.18±0.73 | <0.05 |  |
| Kidney, mg/TL | 8.94±0.76 | 8.55±0.97 | NS |  |
| **Echocardiography** |  |  |  |  |
| Heart rate, beats/min | 356±17 | 362±14 | NS | |
| WTd, mm | 0.73±0.04 | 0.85±0.05 | NS(=0.054) | |
| LVDd, mm | 4.03±0.06 | 4.51±0.12 | <0.05 | |
| LVDs, mm | 2.42±0.41 | 3.09±27 | <0.05 | |
| FS, % | 40.2±3.8 | 31.7±5.5 | <0.05 | |
| E/A ratio | 1.78±0.24 | 1.25±0.35 | <0.05 | |

Results are means±S.D. Abbreviations: TL, tibia length; LV, left ventricle; RV, right ventricle; WTd: wall thickness at diastole; LVDd and LVDs: LV dimension at diastole or systole; FS: fractional shortening; E/A: early- or atrial wave of LV diastolic filling flows. NS: not significant.
